# Nicotinamide adenine dinucleotide replenishment rescues colon degeneration in aged mice

**DOI:** 10.1038/sigtrans.2017.17

**Published:** 2017-07-07

**Authors:** Xudong Zhu, Weiyan Shen, Ying Wang, Amit Jaiswal, Zhenyu Ju, Qinsong Sheng

**Affiliations:** 1 Institute of Ageing Research, Hangzhou Normal University School of Medicine, Hangzhou, China; 2 Leibniz Institute for Age Research-Fritz Lipmann Institute (FLI), Jena, Germany; 3 Department of Anorectal Surgery, the First Affiliated Hospital of Zhejiang University, Hangzhou, China

## Abstract

Susceptibility of gastrointestinal dysmotility increases with age-associated colonic degeneration. A paucity of remedies reversing colonic degeneration *per se* hinders the fundamental relief of symptoms. Here we discovered the correlation between colon degeneration and altered nicotinamide adenine dinucleotide (NAD) level in aged mice. Compared to 3-month-old young controls, 2-year-old mice showed a spectrum of degenerative colonic phenotypes and exhibited a significant elongated transit time and slowed stool frequency in the context of Lomotil-induced slow-transit constipation. Despite upregulated colonic tryptophan hydroxylases expression, serotonin release and expression of colon-predominant type IV serotonin receptor, reduced viability of interstitial cells of Cajal while enhanced aquaporins (Aqp1, 3 and 11) led to a less colonic motility and increased luminal dehydration in aged mice. Notably, this colonic degeneration was accompanied with reduced key NAD^+^-generating enzyme expression and lowered NAD^+^/NADH ratio in aged colon. Three-month continuous administration of beta nicotinamide mononucleotide, a NAD^+^ precursor, elevated colonic NAD^+^ level and improved defecation in aged mice. In contrast, pharmacological inhibition of nicotinamide phosphoribosyltransferase, the rate-limiting enzyme for NAD^+^ biosynthesis, induced a reduction in colonic NAD content and impaired gastrointestinal function in young mice. Taken together, these findings suggest the beneficial effect of NAD^+^ in maintaining colonic homoeostasis and reactivating NAD^+^ biosynthesis may represent a promising strategy to counteract age-related gastrointestinal degeneration.

## Introduction

The intestinal function declines with advancing age thereby causing an increasing incidence of gastrointestinal dysmotility in the geriatric population.^1,2,3,4,5^ Chronic constipation, especially the slow-transit constipation (STC), represents one of the most common gastrointestinal disorders in China and the western countries. Multifactorial aetiologies contribute to its initiation and development, including dysregulated enteric nervous system,^6,7^ altered gut microbiota composition,^8,9^ aberrant neurohormonal secretion,^10,11,12,13^ food pyramid and other patho-physiological abnormalities.^14,15,16,17^ Although anti-constipation strategies are continuously explored and updated,^18^ the morbidity of constipation irreversibly augments and this partly stems from an age-related rise in prevalence of constipation^19,20,21^ along with the accelerated global ageing. Using human specimens or animal models, many signalling pathways regulating gastrointestinal function were found altered during aging, such as neurokinin 2-mediated attenuation of tachykinin signalling,^15^ and serotonergic signalling abnormalities.^22^ Besides, the morphology and number of villus and crypts, the secretion of anti-inflammatory cytokines and serotonin, the (re-)absorption of nutrients and fluids in colon are all altered compared to those of young. Although there is a lack of information regarding the precise mechanisms of ageing that leads to abnormalities in gastrointestinal motility, rejuvenating the ageing colon holds the promise to relief age-related colonic inertia.

Nicotinamide adenine dinucleotide (NAD) is one of the key coenzymes regulating many metabolic pathways, which is synthesized from extracellular precursors in the form of dinucleotides through two pathways, namely *de novo* pathway and salvage pathway.^23,24^ Recently, mounting evidence suggests that NAD biosynthesis declines with age^25,26,27, 28^ and therefore serving as a hallmark of senescence.^29^ In contrast, repletion of NAD to aged mice restores mitochondrial and stem cell function, but also rejuvenating tissue and extending lifespan.^30,31,32^ Moreover, in mammals NAD^+^ also plays a pivotal role in modulating circadian clock,^33^ hepatosteatosis^34^ and other age-related metabolic complications.^35^ Although significant research has been carried out on various organs, colon remains to be the one of less studied organ during ageing and further the effect of ageing on colonic function is not fully understood. One notion that NAD functions as a purinergic, inhibitory neurotransmitter remains controversial.^36,37,38^ Nonetheless, there is a potential association between age-related alterations in intestinal motility and NAD level, however, it remains to be clarified whether declined NAD is a driver of the degeneration of normal colonic function, and whether repletion of NAD could rescue colonic degeneration of the aged.

To this end, we delineated the age-related alterations in the colon of young and old mice. In current STC models, we found a longer transit time and less faecal output in the aged mice partially due to reduced interstitial cells of Cajal (ICC) availability and augmented Aqp-mediated fluid reabsorption, although the Tph-5-HT-SR4 axis was more activated in aged colon compared to that of young controls. Intriguingly, age-related colon defect links to attenuated NAD^+^ bioavailability. By pharmacological activation or inhibition of NAD^+^ biosynthesis, we uncovered the protective role of NAD^+^ in aged colon both *in vitro* and *in vivo*. Our results demonstrated that NAD^+^ replenishment improved defecation in the aged mice and thus may represent a novel approach to protect against aging-related constipation.

## Materials and methods

### Animal

All procedures involving experimental animals were conducted in full accordance with approval by the Animal Care and Use Committee of Hangzhou Normal University. Young (3-month-old) and old (22 to 25-month-old) C57Bl6J mice were maintained in a temperature-controlled room (22±1 °C) on a 12-h light/dark cycle with *ad libitum* access to food and water.

### Chemicals and antibodies

Beta nicotinamide mononucleotide (β-NMN) (BTO5) was purchased from BONTAC (Shenzhen, China). Nicotinamide phosphoribosyltransferase (Nampt) inhibitor (GMX1778) was from Selleck (Shanghai, China). Lomotil (Atropine-diphenoxylate) was purchased from Kangpu Pharmaceutical Ltd Co (Changzhou, China). Antibodies against serotonin transporter (ab181034), tryptophan hydroxylase (ab52954), Lgr5 (ab75850) and Nampt (ab45890) were purchased from Abcam (Cambridge, MA, USA). SR4 (sc-376158), p15/16 (sc-377412) and goat anti-mouse IgG-FITC (sc-2010) were purchased from Santa Cruz Biotechnology (Dallas, TX, USA). APC anti-mouse CD117 (c-kit) (135108) was purchased from BioLegend (San Diego, CA, USA). Proliferating cell nuclear antigen (2586), GAPDH (5174) and Senescence β-Galactosidase Staining Kit (9860) were purchased from Cell Signaling Technology (Danvers, MA, USA). Goat anti-mouse IgG (31430), goat anti-rabbit IgG (31460) and TRIzol Reagent (15596-018) were purchased from Invitrogen (Carlsbad, CA, USA). EvaGreen Supermix (172-5201AP) was purchased from Bio-Rad Laboratories Inc (Hercules, CA, USA). PrimeScript first strand cDNA Synthesis Kit (6110A) was purchased from TaKaRa Bio Inc (Dalian, China). Nuclear Fast Red solution (N3020) and all other chemicals were purchased from Sigma-Aldrich (St Louis, MO, USA), unless otherwise stated.

### Establishment of slow-transit constipation animal model

The atropine-diphenoxylate (Lomotil) was used to induce slow-transit constipation (STC) in mice according to previous reports^39,40^ with minor modification. Both young and old mice were assigned into two groups receiving either placebo (distilled water) or Lomotil at a dose of 5 mg kg^−1^ per day. STC group were gavaged with 100 μl of distilled water+Lomotil (5 mg kg^−1^) once daily for 14 consecutive days. Vehicle group was treated with the same protocol except using equal amount of distilled water without Lomotil.

### Isolation and culture of primary colon epithelial cells

Primary colon epithelial cells were isolated from freshly excised colon tissue following collagenase type A (Roche, Basel, Switzerland) digestion. Dulbecco’s modified Eagle’s medium (HyClone, Logan, UT, USA) containing 4.5 g l^−1^ glucose, penicillin–streptomycin (100 IU ml^−1^ to 100 μg ml^−1^) and 10% foetal bovine serum (HyClone) was used for primary cell culture.

### Transit time

Transit time was recorded as described^41^ with minor modification. Briefly, mice were fasted overnight and were orally gavaged with 200 μl sterile solution of 6% carmine red (C110713, Aladdin) and 0.5% methylcellulose in water and placed in a new cage with no bedding. Refeeding started immediately post gavage, and all mice started eating within 5 min. Since then mice were monitored every 5 min for production of a red faecal pellet. Transit time was recorded as the total number of minutes elapsed before production of the first red faecal pellet since mice started eating.

### Faecal output and water content

Faecal output was analysed by separately housing each group of mice in a 24-h cycle for continuous 2 weeks. Typically three–five mice of each group were housed in one cage and average pellet output were calculated every 24 h. Three cages of each group of mice were enrolled. Faecal pellets were removed at the end of every 24 h period and overall weight of wet pellet counts was obtained. The pellets were then left to dry at 50 °C for 24 h. Total wet and dry faecal weight was recorded. For the relative faecal water content, the differences between wet and dry faecal weight were divided by wet faecal weight, and this calculation from young mice was normalized as 1.0.

### Colonic methylene blue staining

The staining was performed as previously described.^42^ Briefly, small segments of colonic mucosa (~5×1.0 cm) were pinned out flat and fixed for 2 h in 2% paraformaldehyde in 0.1 M sodium phosphate buffer (pH 7.4) at 4 °C. The fixed tissue was stained for 3–5 min in 0.2% methylene blue in 0.1 M sodium phosphate buffer (pH 7.4) and rinsed in fresh phosphate buffer at 4 °C for 30–60 min to allow more even distribution of the blue stain. The intact mucosa segments were placed luminal side up on microscope slides and observed with a low magnification (×4 objective lens and a ×10 or ×15 ocular lens).

### Senescence β-galactosidase staining

The fresh isolated colons were fixed in fixation solution containing 2% formaldehyde and 0.2% glutaraldehyde in phosphate-buffered saline (PBS) for 1 h, rinsed with PBS three times and then transferred to 30% sucrose and fixed for 24 h. Then after, the tissues were embedded in Tissue-Tek O.C.T. Compound (Sakura Finetek, Torrance, CA, USA) for cutting to 6-μm-thick sections. Cryosections were fixed with 1× fixation buffer for 2 min, rinsed with PBS three times and incubated overnight at 37 °C with the staining mixture supplied from the Senescence β-Galactosidase Staining Kit (Cell Signal, Danvers, MA, USA). The sections were counterstained with Nuclear Fast Red solution for nuclei labelling. Finally, the slides were scanned and photographed using a panoramic MIDI system from 3DHISTECH (Budapest, Hungary).

### Immunoblotting

Total proteins were extracted from colon tissues using RIPA buffer (Applygen Technologies Inc, Beijing, China) supplemented with phosphatase and protease inhibitors (Roche). Equal amounts of proteins were separated by SDS-PAGE, and then transferred to polyvinylidene fluoride membranes (Millipore, Billerica, MA, USA). The membranes were reacted with primary antibody followed by secondary antibody. The immunoreactive bands were detected by Immun-Star WesternC chemiluminescence solutions (Bio-Rad Laboratories Inc).

### Immunohistochemistry and immunofluorescence staining

For immunohistochemical staining, the fresh isolated colons were fixed in 4% paraformaldehyde solution for 24–48 h, embedded in paraffin, then cut to 6 μm and mounted on glass slides. The slides were counterstained by haematoxylin (4 min), rinsed in running water (5 min), incubated with 1% eosin (aqueous, 1 min) and rinsed with water until slide runs clear. Tissues were dehydrated sequentially with 70, 95 and 100% ethanol, immersed in xylene and cover-slipped using a mounting medium, and images were captured using a panoramic MIDI system from 3DHISTECH.

For immunofluorescence staining, sections were deparaffinized, hydrated, washed, antigen unmasked and then blocked in 10% normal goat serum for 1 h, following with incubation overnight at 4 °C with SR4 or Nampt antibody. After washing three times in PBS with 1.5% normal blocking serum, the slides were incubated with goat anti-mouse IgG-FITC for 1 h at room temperature. The slides were then mounted by using Vectorshield Mounting Medium supplemented with 1.5 μg ml^−1^ diamidino-2-phenylindole (H-1200; Vector Laboratories, Burlingame, CA, USA). Immunofluorescent images were acquired using a senior upright Axio imager M2 fluorescence microscope from Carl Zeiss (Jena, Germany).

### Quantitative polymerase chain reaction

Transcriptional expression levels of targeted genes in colon were assayed by quantitative polymerase chain reaction using primer sequences listed in Supplementary Table 1. Fresh excised colon tissues were snap frozen in liquid nitrogen and stored at −80 °C. Total RNA was extracted using Trizol reagent (Life Technologies, Carlsbad, CA, USA) according to the manufacturer’s instructions. Reverse transcription and quantitative polymerase chain reaction were performed using the PrimeScript first strand cDNA Synthesis Kit (TaKaRa Bio Inc) and EvaGreen Supermix (Bio-Rad Laboratories Inc) as per the manufacturers’ instructions. The cycle threshold value determined for each RNA was normalized to the β-actin content to indicate relative RNA level.

### Serotonin measurement

Whole colon was excised longitudinally and rinsed in cold PBS for five times to remove faeces. Longitudinal slim strip (~20 mg) of colon was cut for quantification. Colonic intracellular serotonin (5-HT) levels were measured using a commercial available ELISA kit (ADI-900-175, Enzo Life Sciences, Farmingdale, NY, USA) as per the manufacturers’ instructions.

### Quantification of NAD^+^/NADH ratio

Whole colon was excised longitudinally and rinsed in cold PBS for five times to remove faeces. Longitudinal slim strip (~20 mg) of colon was cut for quantification. Tissue NAD^+^/NADH ratio was analysed with a commercial NADH/NAD quantification kit (K337-100; Biovision, San Francisco, CA, USA) as per the manufacturers’ instructions.

### Flow cytometry

For flow cytometry analysis, APC c-Kit-labelled cell suspensions were analysed with an LSRFortessa cell analyser (BD Biosciences, Franklin Lakes, NJ, USA). Rectangular regions were selected to define clusters with pink (presumed ICC).

### Statistics

The data are represented as mean values±s.e.m. Student’s *t*-tests or one-way ANOVA post Newman–Keuls multiple comparison tests were used where appropriate. *P*<0.05 was considered significant.

## Results

### Defecation difficulty is accompanied with age-associated colonic degeneration

**Figure 1:**
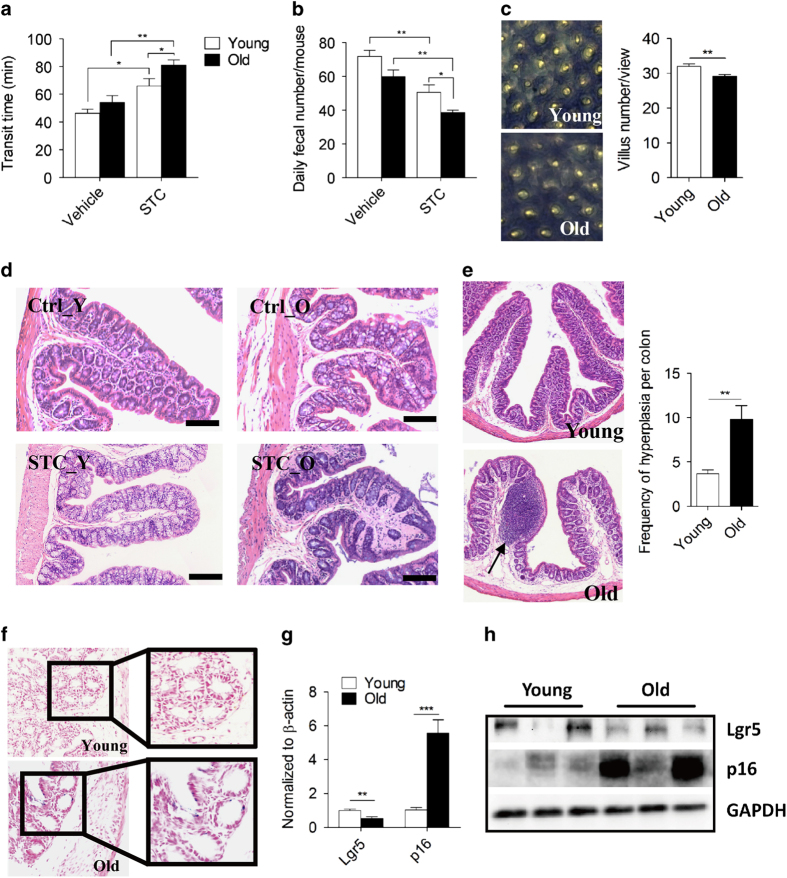
Defecation difficulty is accompanied with age-associated colonic degeneration. (**a**) Total transit time of young and old mice treated with or without Lomotil (*n*=6). Data were presented as mean±s.e.m. *P<0.05, **P<0.01. (**b**) Daily faecal number of young and old mice treated with or without Lomotil (*n*=13–16). Data were presented as mean±s.e.m. *P<0.05, **P<0.01. (**c**) Representative images of methylene blue staining indicate decreased villus number in old colon. Villus numbers were counted in randomly selected six areas for each section. **P<0.01. (**d**) Representative images of haematoxylin and eosin staining from young and old colon treated with or without Lomotil. Bar=100 μm. (**e**) Representative images of haematoxylin and eosin staining from young and old colon and calculation of hyperplasia (*n*=6). Arrow denotes the occurrence site of hyperplasia. Data were presented as mean±s.e.m. **P<0.01. (**f**) Representative images of senescence-associated β-galactosidase (SA-β-gal) staining in cryosections from young and old colons. (**g**) Relative Lgr5 and cdkn2a (p16) mRNA expression in young and colons (*n*=5–9). β-actin was used to normalize data. Data were presented as mean±s.e.m. **P<0.01, ***P<0.001. (**h**) Immunoblotting images of Lgr5 and p16 protein levels in young and old colons.

We compared defecation status of 3-month-old C57BL/6 (young) and 24-month-old (old) mice with identical breeding conditions. Mice body mass, food and drink intake were all similar between the two cohorts. To test whether the effect of (atropine-diphenoxylate) Lomotil-induced slow-transit constipation (STC) on transit time was age-dependent, both young and old mice were assigned into two groups receiving either placebo (distilled water) or Lomotil at a dose of 5 mg kg^−1^ per day. After 2 weeks of gavage, no statistical difference of transit time between young and old mice was seen in vehicle group, but there was a significant elongated transit time in STC group versus that of vehicle group, as well as old mice treated with STC compared to that of young mice (Figure 1a). No sex-related differences were observed in current study (data not shown). The daily faecal output was significantly less in STC-treated groups in comparison to young groups, with an augmented reduction (old: 35.4% versus young: 29.6%) in old group upon Lomotil treatment (Figure 1b). To evaluate whether these amplified defecation difficulty in STC old mice links to age-related colon degeneration, we next examined histological changes between young and old colons. Indeed, a significant reduction in villus number was found in the old colon versus the young colon (Figure 1c). Villus atrophy was seen in both vehicle and STC old mice (Figure 1d), while aged colon exhibited a higher frequency of hyperplasia independent of Lomotil treatment (Figure 1e). Lomotil-induced STC *per se* did not change the villus number or promote hyperplasia (data not shown). In addition, β-galactosidase (SA-β-gal) assay revealed more positive stains in aged colon while almost none in the young colon (Figure 1f), suggesting an increased incidence of age-associated colonic senescence. This was further confirmed by decreased intestinal stem cell marker Lgr5 and increased cell senescence marker cdkn2a (p16) seen in aged colon (Figures 1g and h). Taken together, these data implicate that the defecation difficulty in the aged mice may stem from the degenerative colonic phenotypes.

### Tph-5-HT-SR4 signalling axis is activated in aged colon

**Figure 2:**
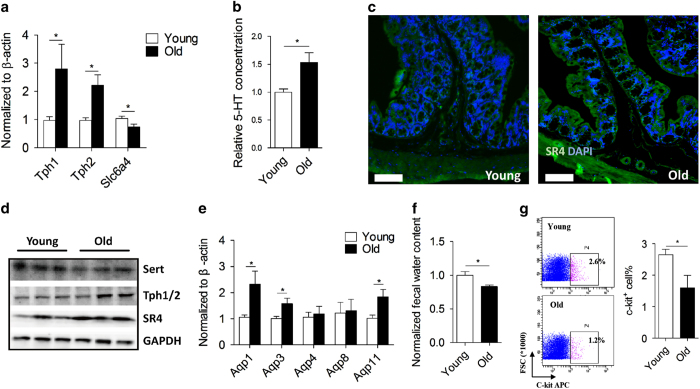
Decreased faecal water content and number of ICC contributes to defecation difficulty in aged mice. (**a**) Relative Tph1, Tph2 and Slc6a4 (sert) mRNA expression in young and colons (*n*=8–12). β-actin was used to normalize data. Data were presented as mean±s.e.m. *P<0.05. (**b**) Relative serotonin (5-HT) release in young and colons (*n*=4). *P<0.05. (**c**) Representative fluorescent images of SR4 expression in young and old colons. Bar=100 μm. (**d**) Immunoblotting images of Sert, Tph1/2 and SR4 protein levels in young and old colons. (**e**) Relative aquaporins mRNA expression in young and colons (*n*=4). β-actin was used to normalize data. Data were presented as mean±s.e.m. *P<0.05. (**f**) Relative faecal water content in young and old mice. Data were presented as mean±s.e.m. *P<0.05. (**g**) Representative flow cytometry plots and quantification of ICC (c-kit positive% gated in pink in the rectangle) in young and old colons (*n*=4). Data were presented as mean±s.e.m. *P<0.05.

We next investigated the molecular mechanism linking defecation difficulty with age-associated colonic degeneration. Given serotonin signalling pathway plays a crucial role in regulating gut motility and defecation,^9,12^ we examined the serotonin release and downstream signalling in young and aged mice. Transcriptional expression of tryptophan hydroxylase 1 (Tph1), the predominant serotonin synthesis enzyme, together with Tph2, were upregulated, while serotonin reuptake transporter Slc6a4 (Sert) was downregulated in old colon in comparison to young colon (Figure 2a). Consistent with upregulated Tph expression, a higher serotonin level (Figure 2b) in parallel with enhanced type IV serotonin receptor (SR4) expression (Figure 2c) were seen in old colons. These results were further confirmed by immunoblotting analysis (Figure 2d), suggesting that the Tph-5-HT-SR4 signalling axis is activated in aged colon.

### Decreased faecal water content and number of ICC in aged mice

Given that increased 5-HT release led to enhanced aquaporin-3 (Aqp3) expression,^43^ which in turn promoted luminal dehydration, we speculated that increased 5-HT in aged colon may lead to enhanced luminal dehydration via increasing local aquaporins expression. Indeed, expression levels of Aqp1, 3 and 11 were upregulated in aged colon while Aqp4 and 8 were unchanged (Figure 2e), leading to a ~17% faecal water content reduction compared to young patterns (Figure 2f). In identifying further correlation to defecation difficulty in aged mice, the intestinal pacemakers, ICC were considered as a possible target due to the ability to control enteric nervous system.^44^ Freshly prepared young and old colonic cells were stained with antibodies against ICC marker c-kit, and flow cytometry analysis displayed a significant reduction of c-kit positive population in aged colonocytes (Figure 2g; young: 2.65±0.17% versus old: 1.60±0.39%). Together, these data suggest that reduced viability of ICC while enhanced aquaporins (Aqp1, 3 and 11) may contribute to a less colonic motility and increased luminal dehydration in aged mice.

### NAD regulates colonic function and homoeostasis

**Figure 3:**
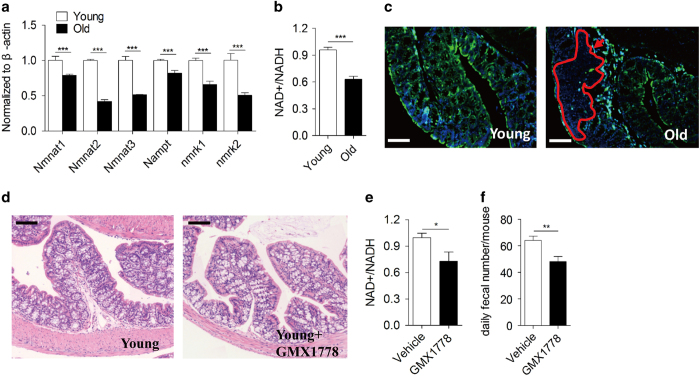
NAD regulates colonic function and homoeostasis. (**a**) Relative mRNA expression of salvage NAD biosynthetic enzymes in young and colons (*n*=3). β-actin was used to normalize data. Data were presented as mean±s.e.m. ***P<0.001. (**b**) Colonic NAD^+^/NADH ratio was analysed in young and colons (*n*=4). ***P<0.001. (**c**) Representative fluorescent images of Nampt expression in young and old colons. Bar=100 μm. Irregularly dotted area in red denotes the occurrence site of hyperplasia. (**d**) Representative images of haematoxylin and eosin staining from young colons treated with or without specific Nampt inhibitor GMX1778. Bar=100 μm. (**e**) Colonic NAD^+^/NADH ratio was analysed in young mice treated with or without Nampt inhibitor GMX1778 (*n*=5). *P<0.05. (**f**) Daily faecal number of young mice treated with or without specific Nampt inhibitor GMX1778 (*n*=6). **P<0.01.

**Figure 4:**
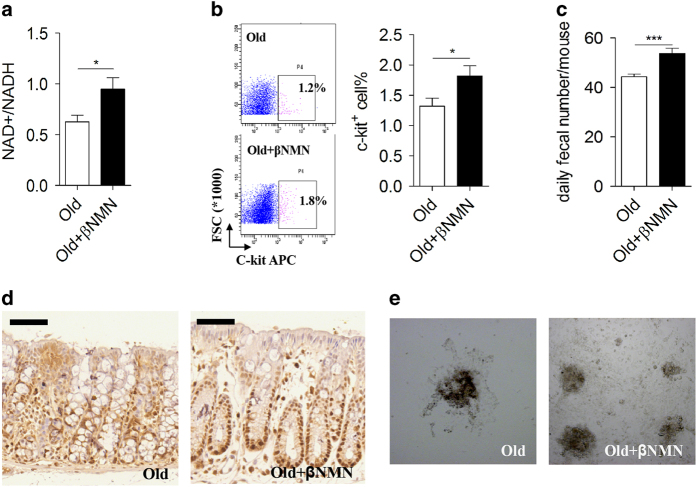
NAD repletion improves colonic function in aged mice. (**a**) Colonic NAD^+^/NADH ratio was analysed in old mice treated with or without β-NMN for 3 months (*n*=5). *P<0.05. (**b**) Representative flow cytometry plots and quantification of ICC (c-kit positive% gated in pink in the rectangle) in old mice treated with or without β-NMN (*n*=5). *P<0.05. (**c**) Daily faecal number of aged mice treated with or without β-NMN (*n*=6). ***P<0.001. (**d**) Representative images of proliferating cell nuclear antigen-stained colonic sections from aged mice treated with or without β-NMN. Bar=50 μm. (**e**) Representative images of primary cultured colonic epithelial cells from aged mice treated with or without β-NMN, on day 7.

Since several lines of evidence demonstrate that oxidized form of NAD^+^ or NAD^+^/NADH ratio is reduced in various age-related pathologies as well as during ageing process,^45,46^ we next assessed the major NAD^+^-generating enzyme expression in the salvage pathway to test whether a reduction in these enzymes accounts for the degeneration in the aged colon. Remarkably, transcription of nicotinamide mononucleotide adenylyltransferase 1, 2 and 3 (Nmnat1, 2 and 3), Nampt as well as nicotinamide riboside kinase 1 and 2 (Nmrk1 and 2) were all downregulated in old colon versus that in young colon (Figure 3a), which was further evidenced as aged colon exhibited a significantly lowered NAD^+^/NADH ratio (Figure 3b). Interestingly, almost no positive immunofluorescent staining of Nampt was seen in those hyperplastic tissues from aged colon (Figure 3c), suggesting that lowered NAD^+^ level may correlate with vulnerability of hyperplasia/tumorigenesis. To further explore the correlation between NAD^+^ level and colonic function, we administered the Nampt inhibitor GMX1778 to 2-month-old young mice intraperitoneally twice daily for 4 weeks. Injection of GMX1778 to young mice led to an ageing colon phenotype, including thinning of colonic muscle layer and villus atrophy (Figure 3d), with concurrent reductions in NAD content and faecal output (Figures 3e and f). In contrast, 3-month continuous administration of β-NMN, an NAD^+^ precursor, restored colonic NAD^+^ level to that of young mice (Figure 4a) and improved colon function. Specifically, old mice receiving β-NMN favoured an increased colonic c-kit^+^ population (Figure 4b) and significantly improved faecal output (Figure 4c). In addition, enhanced proliferation was seen in the isolated colon epithelial cells (Figure 4d) as well as proliferating cell nuclear antigen-stained from aged mice receiving β-NMN (Figure 4e). Taken together, these data indicate that NAD^+^ may serve as a regulator in colon homoeostasis during ageing and repletion of NAD^+^ is able to improve colon function.

## Discussion

Colon ageing featured by altered morphology, dysregulated enteric nervous system, decreased secretion and propulsive motility, and other phenotypes, causes multiple gastrointestinal disorders in the elderly. In this study, we demonstrated that aged mice exhibited a spectrum of colon ageing phenotypes such as longer transit time and less faecal output partially due to reduced ICC availability and augmented aquaporin-mediated fluid reabsorption. Inhibiting NAD biosynthesis in young mice exhibited similar phenotype of aged colon. Intriguingly, these age-related colon defects could be partially rescued by NAD^+^ repletion, thus, our results light up the veiled connection between NAD^+^ and colon ageing.

Chronic constipation represents one of the most common problems among older people, exploration of its etiopathogenesis and emerging strategies of anti-constipation continuously accelerate the relief of symptom, however, slow progress has been made due to the massive heterogeneity among humans and lack of suitable drugs that can fundamentally rejuvenate the ageing colon, plus the inevitable global ageing. Unlike human gastrointestinal studies puzzled by mixed genetic backgrounds, different geographic allocation and lifestyles, mice of high homogeneity fed standard chow and housed in an environment of constant temperature and humidity allow us to the explore the mechanisms underlying the age-related intestinal changes and its relevance to gastrointestinal disorders. By comparing young (3-month-old) with old (24-month-old) mice, we observed significant colon degeneration in old mice, including villus atrophy, aberrant hyperplasia, increased senescence markers and defecation difficulty. Of note, the expression of Lgr5, the intestinal stem cell marker, was markedly reduced in old colon, which is thought to impair colon function by limiting the differentiation and proliferation of specific cell types regulating colon motility such as enterochromaffin cells,^9^ ICC,^44^ epithelial cells^47^ and so on.

The Tph-5-HT-SR4 axis has been long studied in colon motility.^48,49,50^ Elevation of Tph expression and activity promotes the biosynthesis of 5-HT. Increased 5-HT concentration and binding to its receptor in colon further enhances bowel movement and accelerates colon transit. Abnormal Tph-5-HT-SR4 signalling has been found in diverticular disease,^50^ inflammatory bowel disease^51^ and irritable bowel syndrome.^52^ Previous observations have shown increased Tph1 activity, EC cell 5-HT content and 5-HT release, but not decreased sert activity in patients with chronic constipation.^11,53,54^ In agreement, our results confirmed that aged colon exhibited an activated Tph-5-HT-SR4 signalling pathway with a decreased sert expression. Given activated Tph-5-HT-SR4 signalling leads to an enhanced intestinal propulsion, it seems paradoxical that aged colon exhibited a higher 5-HT level with a decreased stool output. One possible explanation is that 5-HT receptor is desensitized upon long-term exposure of high 5-HT level, which leads to visceral hyposensitivity thereby a resulting reduction in stool frequency. Consistently, acutely increasing serotonergic activity with the selective serotonin reuptake inhibitor showed no effect on rectal motor function.^55,56^ In addition, serotonin was found to elevate aquaporin-3 expression in the colon using a morphine-induced constipation, we also found decreased faecal water content and shrinking number of ICC in aged mice, suggesting that altered 5-HT signalling as a crucial driver to defecation problem in the aged.

Many studies have linked NAD with colorectal cancer,^23^ however, currently no direct evidence confirm the association between NAD level and defecation problem, although susceptibility of constipation increases with age concurrent with ageing-related decreasing NAD level.^15^ Besides, a previous drug safety and efficacy study^57^ reported that NAD depleting drugs (mainly, Nampt inhibitors) caused various gastrointestinal symptoms, including constipation, suggesting NAD may play a role in regulating colon function. Indeed, our results demonstrated that administration of Nampt inhibitor GMX1778 was able to reduce faecal output and impair colon homoeostasis. In contrast, repletion of NAD via its precursor β-NMN attenuated several colonic ageing phenotypes and improved defecation in old mice. One possible mechanism is that NAD serves as an enteric inhibitory neurotransmitter thereby contributing to neural regulation of colonic motility,^37^ although this remains controversial.^38^ Further study is of great significance to elaborate how NAD involves in the regulation of colonic motility. If so, it remains to be clarified if NAD plays a similar role in other colon dysfunction diseases like inflammatory bowel disease and irritable bowel syndrome.

In summary, our data indicate that ageing-related downregulation of colonic NAD level could be one of the causes of colon degeneration. Such lowered NAD biosynthesis in aged colon in parallel with increased tryptophan-5-HT signalling axis, decreased ICC cells and increased luminal dehydration could be one possible reason leading to the condition of constipation. By replenishing NAD, we observed a significant improvement of faecal output in the old mice, thus opening a novel avenue for the drug design to combat constipation.
