# Supplementary material for: Nicotinamide adenine dinucleotide replenishment rescues colon degeneration in aged mice
Source: Signal Transduct Target Ther. 2017 Jul 7;2:17017–. doi: 10.1038/sigtrans.2017.17 (PMC5657423; doi:10.1038/sigtrans.2017.17)
Supplement: Supplementary Table 1 [file sigtrans201717-s1.docx]

Table 1. Primers used for quantitative real-time PCR.

| **Primer** | **Sequence (5’ - 3’)** |
| --- | --- |
| Lgr5-F | CCTGGACCTCAGTATGAACAAC |
| Lgr5-R | CAGCATAAGCACTTTGAGGC |
| Slc6a4 (Sert)-F | GGCTGAGATGAGGAACGAAG |
| Slc6a4 (Sert)-R | CTATCCAAACCCAGCGTGAT |
| Cdkn2a (p16) -F | GTCGCAGGTTCTTGGTCACT |
| Cdkn2a (p16) -R | CGAATCTGCACCGTAGTTGA |
| Tph1-F | CGTTCCTCTCTTGGCTGAAC |
| Tph1-R | CTGATGGAAGAAAGCAAGCC |
| Tph2-F | CGACCATCCAGGATTTAAGGA |
| Tph2-R | TGACCATATTTATAGCCCATGG |
| Aqp1-F | ACCTGCTGGCGATTGACTAC |
| Aqp1-R | GAAAATCCAGTGGTTTGAGAAG |
| Aqp3-F | TGATGTTTGGCTGTGGCTCC |
| Aqp3-R | TTCAAGTGGGCACCAGACAC |
| Aqp4-F | GTTTGCAATCAATTATACTGGAG |
| Aqp4-R | GTCCAACCCAATATATCCAGT |
| Aqp8-F | CAGCGGTGGACACTTCAACC |
| Aqp8-R | GACTCACCACTTTAGCCAAG |
| Aqp11-F | CTATGCAGGAGGGAGCCTCA |
| Aqp11-R | CATCATCAGCACACCTACAGA |
| Nmnat1-F | GCTCAGATACCATCAGGAGAA |
| Nmnat1-R | TGGGCACACCTGTTGGTTTG |
| Nmnat2-F | CATTCAGATGTTCGAGAGAGC |
| Nmnat2-R | GACTTGACACAAGGCCCTGTT |
| Nmnat3-F | GTGCTGAGGCACCATCACAG |
| Nmnat3-R | ACTCTGGCAGTGCTGCAGAG |
| Nmrk1-F | GAATTGGTGGTGTGACAAACG |
| Nmrk1-R | TCAGACTCTGGCTTGAAGAAGT |
| Nmrk2-F | CATAGGCATTGGAGGGGTGA |
| Nmrk2-R | GGTCCTGGGGCTTGAAGAAG |
| Nampt-F | CTTAAAAGGTAAAGTAGTGACC |
| Nampt-R | CATCGTATTTCTCAAGGATGT |
| b-actin-F | CAGCCTTCCTTCTTGGGTAT |
| b-actin-R | TGGCATAGAGGTCTTTACGG |
